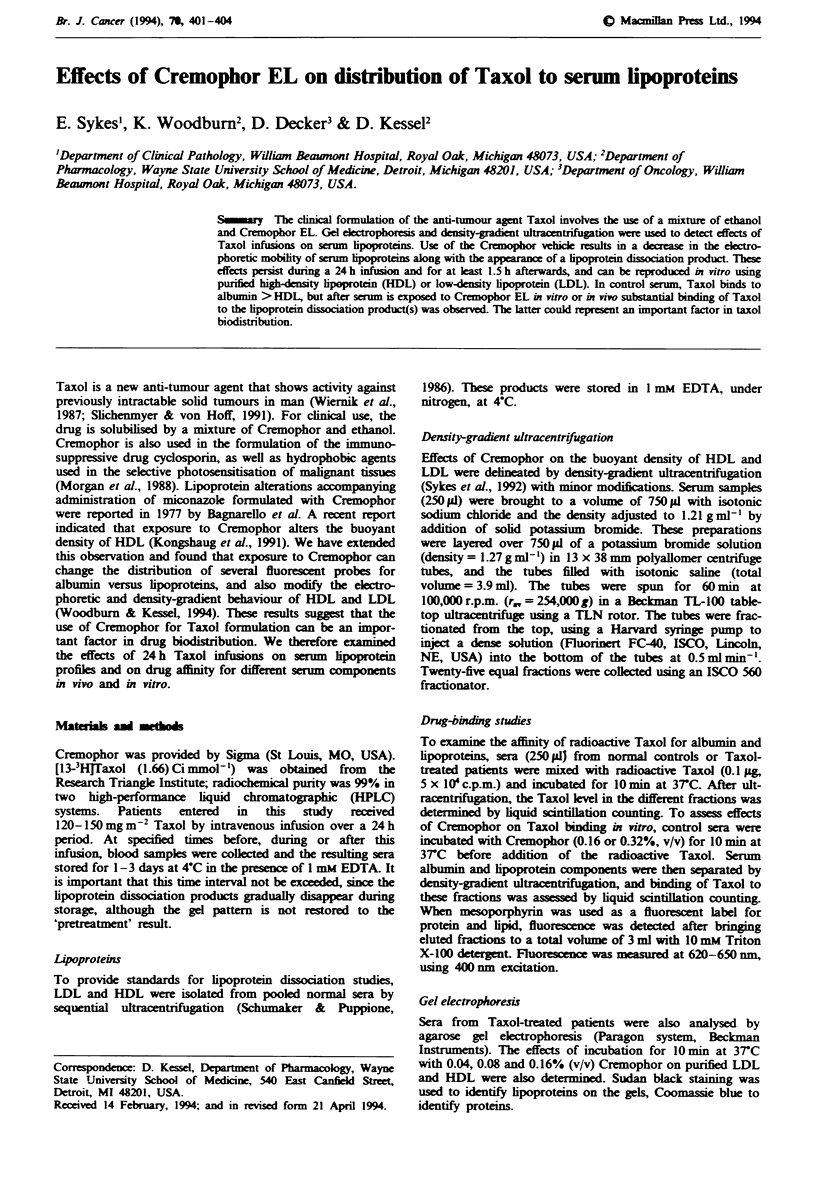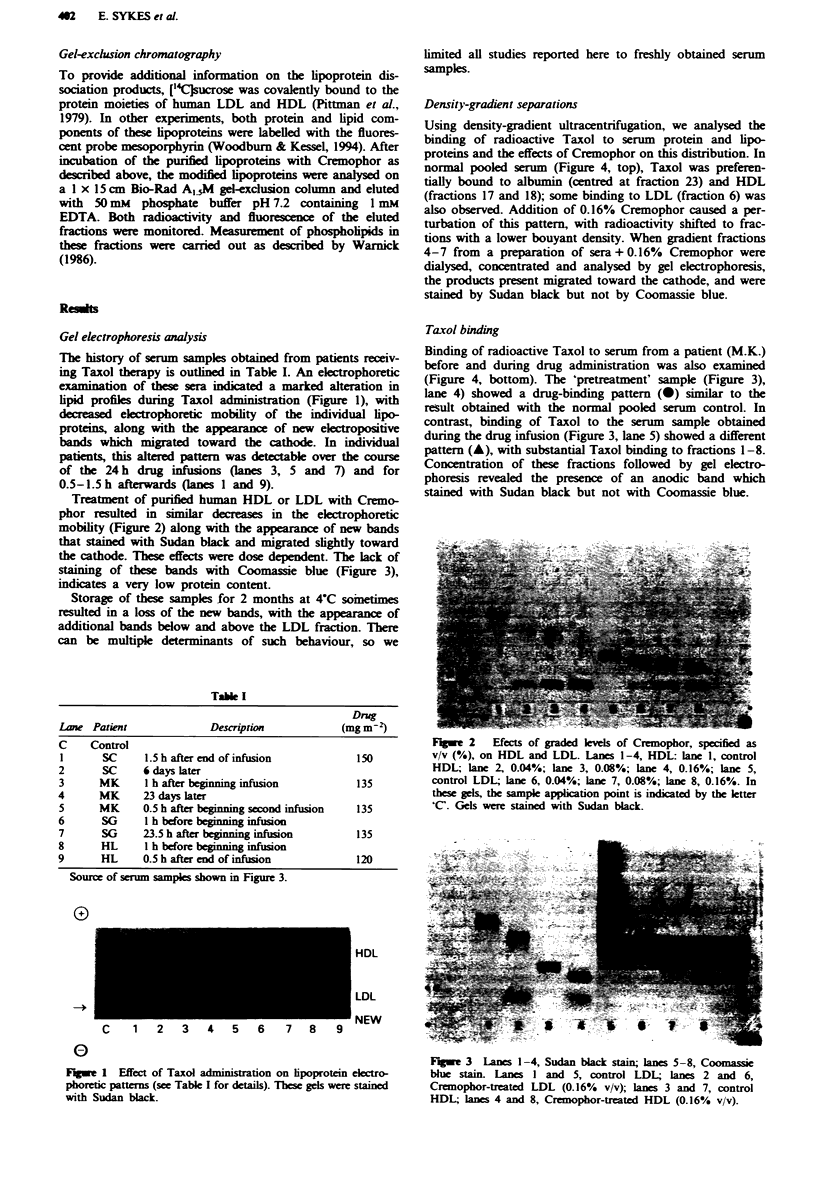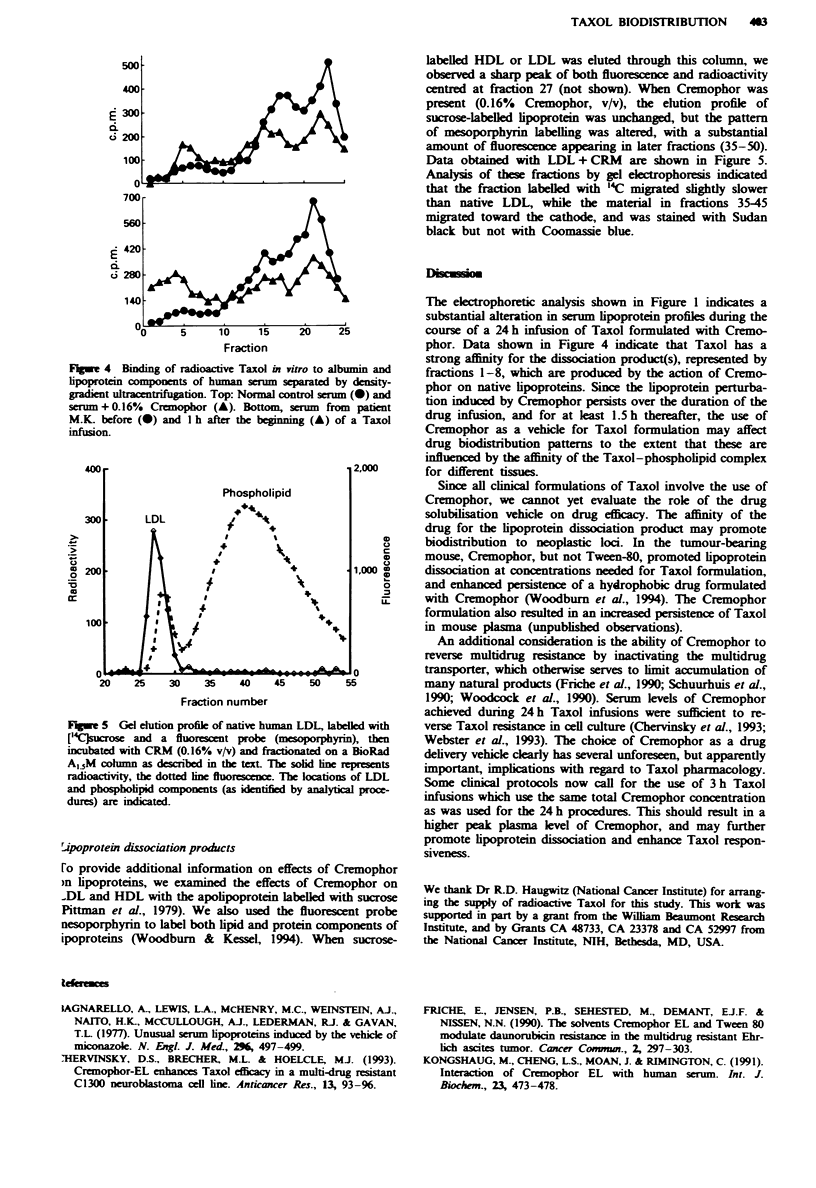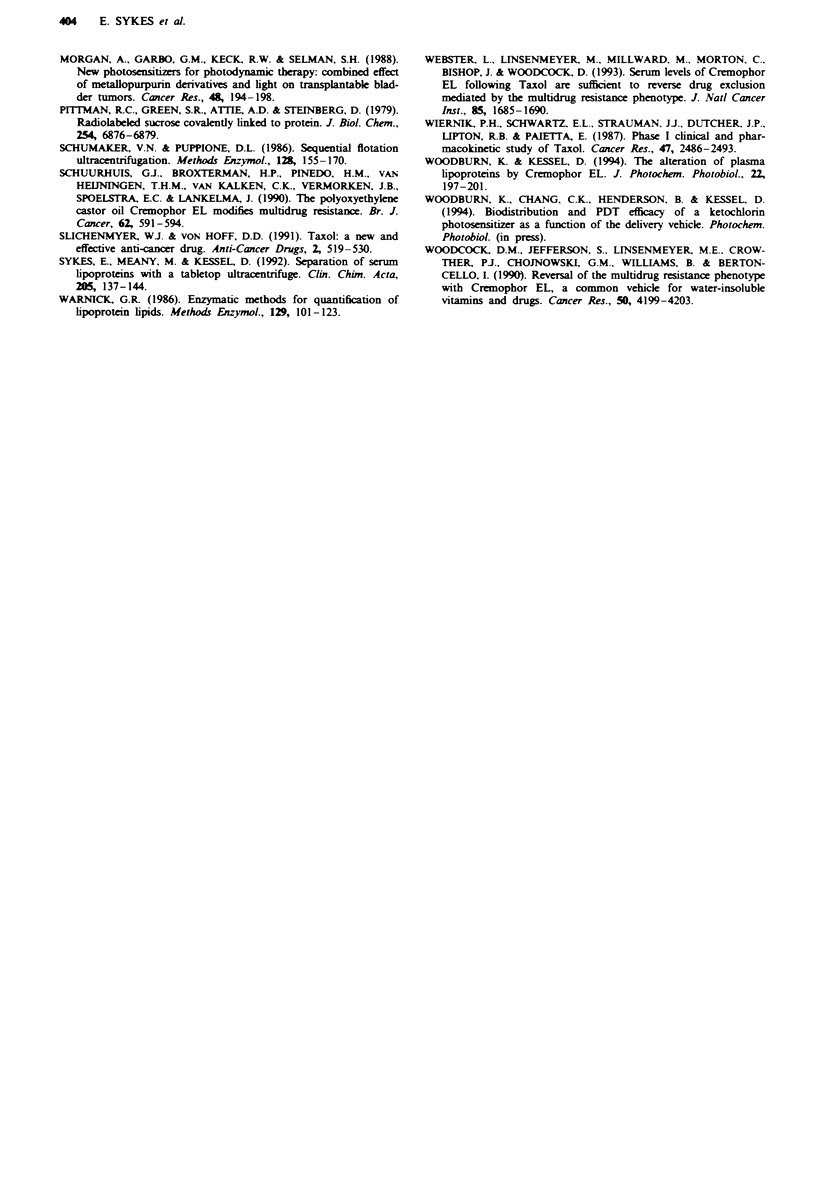# Effects of Cremophor EL on distribution of Taxol to serum lipoproteins.

**DOI:** 10.1038/bjc.1994.317

**Published:** 1994-09

**Authors:** E. Sykes, K. Woodburn, D. Decker, D. Kessel

**Affiliations:** Department of Clinical Pathology, William Beaumont Hospital, Royal Oak, Michigan 48073.

## Abstract

**Images:**


Br. J. Cancer (1994), 70, 401-404 © Macmillan Press Ltd., 1994

### Effects of Cremophor EL on distribution of Taxol to serum lipoproteins

E. Sykes[1], K. Woodburn[2], D. Decker[3] & D. Kessel[2]

[1]*Department of Clinical Pathology, William Beaumont Hospital, Royal Oak, Michigan 48073, USA;* [2]*Department of Pharmacology, Wayne State University School of Medicine, Detroit, Michigan 48201, USA;* [3]*Department of Oncology, William Beaumont Hospital, Royal Oak, Michigan 48073, USA.*

**Summary** The clinical formulation of the anti-tumour agent Taxol involves the use of a mixture of ethanol and Cremophor EL. Gel electrophoresis and density-gradient ultracentrifugation were used to detect effects of Taxol infusions on serum lipoproteins. Use of the Cremophor vehicle results in a decrease in the electrophoretic mobility of serum lipoproteins along with the appearance of a lipoprotein dissociation product. These effects persist during a 24 h infusion and for at least 1.5 h afterwards, and can be reproduced *in vitro* using purified high-density lipoprotein (HDL) or low-density lipoprotein (LDL). In control serum, Taxol binds to albumin > HDL, but after serum is exposed to Cremophor EL *in vitro* or *in vivo* substantial binding of Taxol to the lipoprotein dissociation product(s) was observed. The latter could represent an important factor in taxol biodistribution.

Taxol is a new anti-tumour agent that shows activity against previously intractable solid tumours in man (Wiernik *et al.*, 1987; Slichenmyer & von Hoff, 1991). For clinical use, the drug is solubilised by a mixture of Cremophor and ethanol. Cremophor is also used in the formulation of the immunosuppressive drug cyclosporin, as well as hydrophobic agents used in the selective photosensitisation of malignant tissues (Morgan *et al.*, 1988). Lipoprotein alterations accompanying administration of miconazole formulated with Cremophor were reported in 1977 by Bagnarello *et al.* A recent report indicated that exposure to Cremophor alters the buoyant density of HDL (Kongshaug *et al.*, 1991). We have extended this observation and found that exposure to Cremophor can change the distribution of several fluorescent probes for albumin versus lipoproteins, and also modify the electrophoretic and density-gradient behaviour of HDL and LDL (Woodburn & Kessel, 1994). These results suggest that the use of Cremophor for Taxol formulation can be an important factor in drug biodistribution. We therefore examined the effects of 24 h Taxol infusions on serum lipoprotein profiles and on drug affinity for different serum components *in vivo* and *in vitro*.

### Materials and methods

Cremophor was provided by Sigma (St Louis, MO, USA). [13-$^3$H]Taxol (1.66) Ci mmol$^{-1}$) was obtained from the Research Triangle Institute; radiochemical purity was 99% in two high-performance liquid chromatographic (HPLC) systems. Patients entered in this study received 120-150 mg m$^{-2}$ Taxol by intravenous infusion over a 24 h period. At specified times before, during or after this infusion, blood samples were collected and the resulting sera stored for 1-3 days at 4°C in the presence of 1 mM EDTA. It is important that this time interval not be exceeded, since the lipoprotein dissociation products gradually disappear during storage, although the gel pattern is not restored to the 'pretreatment' result.

### *Lipoproteins*

To provide standards for lipoprotein dissociation studies, LDL and HDL were isolated from pooled normal sera by sequential ultracentrifugation (Schumaker & Puppione, 1986). These products were stored in 1 mM EDTA, under nitrogen, at 4°C.

### *Density-gradient ultracentrifugation*

Effects of Cremophor on the buoyant density of HDL and LDL were delineated by density-gradient ultracentrifugation (Sykes *et al.*, 1992) with minor modifications. Serum samples (250 µl) were brought to a volume of 750 µl with isotonic sodium chloride and the density adjusted to 1.21 g ml$^{-1}$ by addition of solid potassium bromide. These preparations were layered over 750 µl of a potassium bromide solution (density = 1.27 g ml$^{-1}$) in 13 × 38 mm polyallomer centrifuge tubes, and the tubes filled with isotonic saline (total volume = 3.9 ml). The tubes were spun for 60 min at 100,000 r.p.m. ($r_{av}$ = 254,000 *g*) in a Beckman TL-100 table-top ultracentrifuge using a TLN rotor. The tubes were fractionated from the top, using a Harvard syringe pump to inject a dense solution (Fluorinert FC-40, ISCO, Lincoln, NE, USA) into the bottom of the tubes at 0.5 ml min$^{-1}$. Twenty-five equal fractions were collected using an ISCO 560 fractionator.

### *Drug-binding studies*

To examine the affinity of radioactive Taxol for albumin and lipoproteins, sera (250 µl) from normal controls or Taxol-treated patients were mixed with radioactive Taxol (0.1 µg, 5 × 10$^4$ c.p.m.) and incubated for 10 min at 37°C. After ultracentrifugation, the Taxol level in the different fractions was determined by liquid scintillation counting. To assess effects of Cremophor on Taxol binding *in vitro*, control sera were incubated with Cremophor (0.16 or 0.32%, v/v) for 10 min at 37°C before addition of the radioactive Taxol. Serum albumin and lipoprotein components were then separated by density-gradient ultracentrifugation, and binding of Taxol to these fractions was assessed by liquid scintillation counting. When mesoporphyrin was used as a fluorescent label for protein and lipid, fluorescence was detected after bringing eluted fractions to a total volume of 3 ml with 10 mM Triton X-100 detergent. Fluorescence was measured at 620-650 nm, using 400 nm excitation.

### *Gel electrophoresis*

Sera from Taxol-treated patients were also analysed by agarose gel electrophoresis (Paragon system, Beckman Instruments). The effects of incubation for 10 min at 37°C with 0.04, 0.08 and 0.16% (v/v) Cremophor on purified LDL and HDL were also determined. Sudan black staining was used to identify lipoproteins on the gels, Coomassie blue to identify proteins.

Correspondence: D. Kessel, Department of Pharmacology, Wayne State University School of Medicine, 540 East Canfield Street, Detroit, MI 48201, USA.
Received 14 February, 1994; and in revised form 21 April 1994.

*Gel-exclusion chromatography*

To provide additional information on the lipoprotein dissociation products, [$^{14}$C]sucrose was covalently bound to the protein moieties of human LDL and HDL (Pittman *et al.*, 1979). In other experiments, both protein and lipid components of these lipoproteins were labelled with the fluorescent probe mesoporphyrin (Woodburn & Kessel, 1994). After incubation of the purified lipoproteins with Cremophor as described above, the modified lipoproteins were analysed on a 1 × 15 cm Bio-Rad $A_{1.5}$M gel-exclusion column and eluted with 50 mM phosphate buffer pH 7.2 containing 1 mM EDTA. Both radioactivity and fluorescence of the eluted fractions were monitored. Measurement of phospholipids in these fractions were carried out as described by Warnick (1986).

### Results

*Gel electrophoresis analysis*

The history of serum samples obtained from patients receiving Taxol therapy is outlined in Table I. An electrophoretic examination of these sera indicated a marked alteration in lipid profiles during Taxol administration (Figure 1), with decreased electrophoretic mobility of the individual lipoproteins, along with the appearance of new electropositive bands which migrated toward the cathode. In individual patients, this altered pattern was detectable over the course of the 24 h drug infusions (lanes 3, 5 and 7) and for 0.5–1.5 h afterwards (lanes 1 and 9).

Treatment of purified human HDL or LDL with Cremophor resulted in similar decreases in the electrophoretic mobility (Figure 2) along with the appearance of new bands that stained with Sudan black and migrated slightly toward the cathode. These effects were dose dependent. The lack of staining of these bands with Coomassie blue (Figure 3), indicates a very low protein content.

Storage of these samples for 2 months at 4°C sometimes resulted in a loss of the new bands, with the appearance of additional bands below and above the LDL fraction. There can be multiple determinants of such behaviour, so we limited all studies reported here to freshly obtained serum samples.

*Density-gradient separations*

Using density-gradient ultracentrifugation, we analysed the binding of radioactive Taxol to serum protein and lipoproteins and the effects of Cremophor on this distribution. In normal pooled serum (Figure 4, top), Taxol was preferentially bound to albumin (centred at fraction 23) and HDL (fractions 17 and 18); some binding to LDL (fraction 6) was also observed. Addition of 0.16% Cremophor caused a perturbation of this pattern, with radioactivity shifted to fractions with a lower bouyant density. When gradient fractions 4–7 from a preparation of sera + 0.16% Cremophor were dialysed, concentrated and analysed by gel electrophoresis, the products present migrated toward the cathode, and were stained by Sudan black but not by Coomassie blue.

*Taxol binding*

Binding of radioactive Taxol to serum from a patient (M.K.) before and during drug administration was also examined (Figure 4, bottom). The 'pretreatment' sample (Figure 3), lane 4) showed a drug-binding pattern (●) similar to the result obtained with the normal pooled serum control. In contrast, binding of Taxol to the serum sample obtained during the drug infusion (Figure 3, lane 5) showed a different pattern (▲), with substantial Taxol binding to fractions 1–8. Concentration of these fractions followed by gel electrophoresis revealed the presence of an anodic band which stained with Sudan black but not with Coomassie blue.

**Table I**

| Lane | Patient | Description | Drug (mg m$^{-2}$) |
|---|---|---|---|
| C | Control | | |
| 1 | SC | 1.5 h after end of infusion | 150 |
| 2 | SC | 6 days later | |
| 3 | MK | 1 h after beginning infusion | 135 |
| 4 | MK | 23 days later | |
| 5 | MK | 0.5 h after beginning second infusion | 135 |
| 6 | SG | 1 h before beginning infusion | |
| 7 | SG | 23.5 h after beginning infusion | 135 |
| 8 | HL | 1 h before beginning infusion | |
| 9 | HL | 0.5 h after end of infusion | 120 |

Source of serum samples shown in Figure 3.

**Figure 1** Effect of Taxol administration on lipoprotein electrophoretic patterns (see Table I for details). These gels were stained with Sudan black.

**Figure 2** Efects of graded levels of Cremophor, specified as v/v (%), on HDL and LDL. Lanes 1–4, HDL: lane 1, control HDL; lane 2, 0.04%; lane 3, 0.08%; lane 4, 0.16%; lane 5, control LDL; lane 6, 0.04%; lane 7, 0.08%; lane 8, 0.16%. In these gels, the sample application point is indicated by the letter 'C'. Gels were stained with Sudan black.

**Figure 3** Lanes 1–4, Sudan black stain; lanes 5–8, Coomassie blue stain. Lanes 1 and 5, control LDL; lanes 2 and 6, Cremophor-treated LDL (0.16% v/v); lanes 3 and 7, control HDL; lanes 4 and 8, Cremophor-treated HDL (0.16% v/v).

**Figure 4** Binding of radioactive Taxol *in vitro* to albumin and lipoprotein components of human serum separated by density-gradient ultracentrifugation. Top: Normal control serum (●) and serum + 0.16% Cremophor (▲). Bottom, serum from patient M.K. before (●) and 1 h after the beginning (▲) of a Taxol infusion.

**Figure 5** Gel elution profile of native human LDL, labelled with [$^{14}$C]sucrose and a fluorescent probe (mesoporphyrin), then incubated with CRM (0.16% v/v) and fractionated on a BioRad $A_{15}M$ column as described in the text. The solid line represents radioactivity, the dotted line fluorescence. The locations of LDL and phospholipid components (as identified by analytical procedures) are indicated.

### *Lipoprotein dissociation products*

To provide additional information on effects of Cremophor on lipoproteins, we examined the effects of Cremophor on LDL and HDL with the apolipoprotein labelled with sucrose Pittman *et al.*, 1979). We also used the fluorescent probe mesoporphyrin to label both lipid and protein components of lipoproteins (Woodburn & Kessel, 1994). When sucrose-labelled HDL or LDL was eluted through this column, we observed a sharp peak of both fluorescence and radioactivity centred at fraction 27 (not shown). When Cremophor was present (0.16% Cremophor, v/v), the elution profile of sucrose-labelled lipoprotein was unchanged, but the pattern of mesoporphyrin labelling was altered, with a substantial amount of fluorescence appearing in later fractions (35–50). Data obtained with LDL + CRM are shown in Figure 5. Analysis of these fractions by gel electrophoresis indicated that the fraction labelled with $^{14}$C migrated slightly slower than native LDL, while the material in fractions 35-45 migrated toward the cathode, and was stained with Sudan black but not with Coomassie blue.

### Discussion

The electrophoretic analysis shown in Figure 1 indicates a substantial alteration in serum lipoprotein profiles during the course of a 24 h infusion of Taxol formulated with Cremophor. Data shown in Figure 4 indicate that Taxol has a strong affinity for the dissociation product(s), represented by fractions 1–8, which are produced by the action of Cremophor on native lipoproteins. Since the lipoprotein perturbation induced by Cremophor persists over the duration of the drug infusion, and for at least 1.5 h thereafter, the use of Cremophor as a vehicle for Taxol formulation may affect drug biodistribution patterns to the extent that these are influenced by the affinity of the Taxol–phospholipid complex for different tissues.

Since all clinical formulations of Taxol involve the use of Cremophor, we cannot yet evaluate the role of the drug solubilisation vehicle on drug efficacy. The affinity of the drug for the lipoprotein dissociation product may promote biodistribution to neoplastic loci. In the tumour-bearing mouse, Cremophor, but not Tween-80, promoted lipoprotein dissociation at concentrations needed for Taxol formulation, and enhanced persistence of a hydrophobic drug formulated with Cremophor (Woodburn *et al.*, 1994). The Cremophor formulation also resulted in an increased persistence of Taxol in mouse plasma (unpublished observations).

An additional consideration is the ability of Cremophor to reverse multidrug resistance by inactivating the multidrug transporter, which otherwise serves to limit accumulation of many natural products (Friche *et al.*, 1990; Schuurhuis *et al.*, 1990; Woodcock *et al.*, 1990). Serum levels of Cremophor achieved during 24 h Taxol infusions were sufficient to reverse Taxol resistance in cell culture (Chervinsky *et al.*, 1993; Webster *et al.*, 1993). The choice of Cremophor as a drug delivery vehicle clearly has several unforeseen, but apparently important, implications with regard to Taxol pharmacology. Some clinical protocols now call for the use of 3 h Taxol infusions which use the same total Cremophor concentration as was used for the 24 h procedures. This should result in a higher peak plasma level of Cremophor, and may further promote lipoprotein dissociation and enhance Taxol responsiveness.

We thank Dr R.D. Haugwitz (National Cancer Institute) for arranging the supply of radioactive Taxol for this study. This work was supported in part by a grant from the William Beaumont Research Institute, and by Grants CA 48733, CA 23378 and CA 52997 from the National Cancer Institute, NIH, Bethesda, MD, USA.

### References

BAGNARELLO, A., LEWIS, L.A., MCHENRY, M.C., WEINSTEIN, A.J., NAITO, H.K., MCCULLOUGH, A.J., LEDERMAN, R.J. & GAVAN, T.L. (1977). Unusual serum lipoproteins induced by the vehicle of miconazole. *N. Engl. J. Med.*, **296**, 497–499.

CHERVINSKY, D.S., BRECHER, M.L. & HOELCLE, M.J. (1993). Cremophor-EL enhances Taxol efficacy in a multi-drug resistant C1300 neuroblastoma cell line. *Anticancer Res.*, **13**, 93–96.

FRICHE, E., JENSEN, P.B., SEHESTED, M., DEMANT, E.J.F. & NISSEN, N.N. (1990). The solvents Cremophor EL and Tween 80 modulate daunorubicin resistance in the multidrug resistant Ehrlich ascites tumor. *Cancer Commun.*, **2**, 297–303.

KONGSHAUG, M., CHENG, L.S., MOAN, J. & RIMINGTON, C. (1991). Interaction of Cremophor EL with human serum. *Int. J. Biochem.*, **23**, 473–478.

MORGAN, A., GARBO, G.M., KECK, R.W. & SELMAN, S.H. (1988). New photosensitizers for photodynamic therapy: combined effect of metallopurpurin derivatives and light on transplantable bladder tumors. *Cancer Res.*, **48**, 194–198.

PITTMAN, R.C., GREEN, S.R., ATTIE, A.D. & STEINBERG, D. (1979). Radiolabeled sucrose covalently linked to protein. *J. Biol. Chem.*, **254**, 6876–6879.

SCHUMAKER, V.N. & PUPPIONE, D.L. (1986). Sequential flotation ultracentrifugation. *Methods Enzymol.*, **128**, 155–170.

SCHUURHUIS, G.J., BROXTERMAN, H.P., PINEDO, H.M., VAN HEIJNINGEN, T.H.M., VAN KALKEN, C.K., VERMORKEN, J.B., SPOELSTRA, E.C. & LANKELMA, J. (1990). The polyoxyethylene castor oil Cremophor EL modifies multidrug resistance. *Br. J. Cancer*, **62**, 591–594.

SLICHENMYER, W.J. & VON HOFF, D.D. (1991). Taxol: a new and effective anti-cancer drug. *Anti-Cancer Drugs*, **2**, 519–530.

SYKES, E., MEANY, M. & KESSEL, D. (1992). Separation of serum lipoproteins with a tabletop ultracentrifuge. *Clin. Chim. Acta*, **205**, 137–144.

WARNICK, G.R. (1986). Enzymatic methods for quantification of lipoprotein lipids. *Methods Enzymol.*, **129**, 101–123.

WEBSTER, L., LINSENMEYER, M., MILLWARD, M., MORTON, C., BISHOP, J. & WOODCOCK, D. (1993). Serum levels of Cremophor EL following Taxol are sufficient to reverse drug exclusion mediated by the multidrug resistance phenotype. *J. Natl Cancer Inst.*, **85**, 1685–1690.

WIERNIK, P.H., SCHWARTZ, E.L., STRAUMAN, J.J., DUTCHER, J.P., LIPTON, R.B. & PAIETTA, E. (1987). Phase I clinical and pharmacokinetic study of Taxol. *Cancer Res.*, **47**, 2486–2493.

WOODBURN, K. & KESSEL, D. (1994). The alteration of plasma lipoproteins by Cremophor EL. *J. Photochem. Photobiol.*, **22**, 197–201.

WOODBURN, K., CHANG, C.K., HENDERSON, B. & KESSEL, D. (1994). Biodistribution and PDT efficacy of a ketochlorin photosensitizer as a function of the delivery vehicle. *Photochem. Photobiol.* (in press).

WOODCOCK, D.M., JEFFERSON, S., LINSENMEYER, M.E., CROWTHER, P.J., CHOJNOWSKI, G.M., WILLIAMS, B. & BERTONCELLO, I. (1990). Reversal of the multidrug resistance phenotype with Cremophor EL, a common vehicle for water-insoluble vitamins and drugs. *Cancer Res.*, **50**, 4199–4203.